# Trends of socioeconomic inequalities in overweight and obesity in children and adolescents in Germany

**DOI:** 10.1093/eurpub/ckac130.076

**Published:** 2022-10-25

**Authors:** J Hoebel, J Waldhauer, M Blume, A Schienkiewitz

**Affiliations:** 1 Unit of Social Determinants of Health, Robert Koch Institute, Berlin, Germany; 2 Unit of Health Behaviour, Robert Koch Institute, Berlin, Germany

## Abstract

**Background:**

Overweight and obesity in early life are risk factors for chronic health conditions in the later life course. Children and adolescents from socioeconomically disadvantaged families are more likely to be overweight or obese than their better-off peers. This study examined post-millennial trends of socioeconomic inequalities in the prevalence of overweight and obesity among young people in Germany.

**Methods:**

Repeated cross-sectional data were used from the German health interview and examination survey for children and adolescents (KiGGS). Overweight and obesity were assessed by body mass index, based on measured height and weight of 3- to 17-year-old participants in KiGGS baseline (2003-06) and KiGGS wave 2 (2014-17). Socioeconomic position (SEP) was measured using a composite index of parental education, occupation and income. The regression-based slope index of inequality (SII) and relative index of inequality (RII) were calculated to estimate the extent of absolute and relative inequalities in the prevalence of overweight and obesity.

**Results:**

The overall prevalence of overweight and obesity among 3- to 17-year-olds in Germany did not change over time and was recently 15.4% and 5.9%, respectively. In both survey waves, overweight and obesity were more prevalent in lower SEP-groups. From 2003-06 to 2014-17, overweight increased in the low-SES group, whereas it tended to decrease in those with higher SES. This trend was concomitant with increasing socioeconomic inequalities in overweight over the observation period (SII from 0.11 to 0.19, p = 0.054; RII from 2.1 to 3.7, p = 0.021). No such trend was found for obesity.

**Conclusions:**

Socioeconomic inequalities in overweight have widened among the young in Germany since the early 2000s. Structural interventions that are effective in preventing and reducing overweight in young people from socially disadvantaged backgrounds are still needed.

**Key messages:**

- Overweight increased among young people from socioeconomically disadvantaged families.
- Socioeconomic inequalities in overweight increased among children and adolescents.

